# Lung Cancer Occurrence—Correlation with Serum Chromium Levels and Genotypes

**DOI:** 10.1007/s12011-020-02240-6

**Published:** 2020-07-09

**Authors:** Piotr Baszuk, Beata Janasik, Sandra Pietrzak, Wojciech Marciniak, Edyta Reszka, Katarzyna Białkowska, Ewa Jabłońska, Magdalena Muszyńska, Monika Lesicka, Róża Derkacz, Tomasz Grodzki, Janusz Wójcik, Małgorzata Wojtyś, Tadeusz Dębniak, Cezary Cybulski, Jacek Gronwald, Bartosz Kubisa, Norbert Wójcik, Jarosław Pieróg, Darko Gajić, Piotr Waloszczyk, Rodney J. Scott, Wojciech Wąsowicz, Anna Jakubowska, Jan Lubiński, Marcin R. Lener

**Affiliations:** 1 Department of Genetics and Pathology, International Hereditary Cancer Center, Pomeranian Medical University in Szczecin, ul. Unii Lubelskiej 1, 71-252 Szczecin, Poland; 2 Biological and Environment Monitoring Department, Nofer Institute of Occupational Medicine, ul.św. Teresy od dzieciątka Jezus 8, 91-348 Łódź, Poland; 3 Read-Gene, Grzepnica, ul. Alabastrowa 8, 72-003 Grzepnica, Dobra(Szczecińska) Poland; 4 Department of Molecular Genetics and Epigenetics, Nofer Institute of Occupational Medicine, ul.św. Teresy od dzieciątka Jezus 8, 91-348 Łódź, Poland; 5 Department of Thoracic Surgery and Transplantation, Pomeranian Medical University in Szczecin, ul. A. Sokołowskiego 11, 70-891 Szczecin, Poland; 6 Independent Laboratory of Pathology, Zdunomed, ul. Energetyków 2, 70-656 Szczecin, Poland; 7 Priority Research Centre for Cancer Research, Innovation and Translation, Hunter Medical Research Institute, New Lambton Heights, Australia; 8 School of Biomedical Sciences and Pharmacy, Faculty of Health and Medicine, University of Newcastle, Newcastle, Australia; 9 Division of Molecular Medicine, Pathology North, John Hunter Hospital, New Lambton, NSW 2305 Australia

**Keywords:** Serum Cr level, DNA variants, Lung cancer occurrence, Detection marker

## Abstract

Lung cancer is the leading cause of cancer-related death worldwide. Exposure to environmental and occupational carcinogens is an important cause of lung cancer. One of these substances is chromium, which is found ubiquitously across the planet. The International Agency for Research on Cancer has classified chromium(VI) as a human carcinogen. The aim of this study was to assess whether serum chromium levels, as well as DNA variants in selected genes involved in carcinogenesis, xenobiotic-metabolism, and oxidative stress could be helpful in the detection of lung cancer. We conducted a study using 218 lung cancer patients and 218 matched healthy controls. We measured serum chromium levels and genotyped ten genetic variants in *ERCC2*, *XRCC1*, *MT1B*, *GSTP1*, *ABCB1*, *NQ01*, *CRTC3*, *GPX1*, *SOD2* and *CAT*. The odds ratios of being diagnosed with lung cancer were calculated using conditional logistic regression with respect to serum chromium level and genotypes. The odds ratio for the occurrence of lung cancer increased with increasing serum chromium levels. The difference between the quartiles with the lowest vs. highest chromium level was more than fourfold in the entire group (OR 4.52, CI 2.17–9.42, *p* < 0.01). This correlation was significantly increased by more than twice when specific genotypes were taken into consideration (*ERCC*–rs12181 TT, OR 12.34, CI 1.17–130.01, *p* = 0.04; *CRTC3*–rs12915189 non GG, OR 9.73, CI 1.58–60.10, *p* = 0.01; *GSTP1*–rs1695 non AA, OR 9.47, CI 2.06–43.49, *p* = < 0.01; *CAT*–rs1001179 non CC, OR 9.18, CI 1.64–51.24, *p* = 0.01). Total serum chromium levels > 0.1 μg/L were correlated with 73% (52/71) of lung cancers diagnosed with stage I disease. Our findings support the role of chromium and the influence of key proteins on lung cancer burden in the general population.

**Electronic supplementary material:**

The online version of this article (10.1007/s12011-020-02240-6) contains supplementary material, which is available to authorized users.

## Introduction

Lung cancer remains the most common cause of death among all cancers, contributing to over a million people annually succumbing to this disease worldwide. In Europe, the average 5-year survival for patients with this cancer is estimated to be around 15% [1]. A characteristic feature of this type of cancer is its late diagnosis when at an advanced stage due to late presentation of symptoms when treatment is no longer possible.

Therefore, it is important to improve the possibility of early detection through effective screening tests, which would result in a reduction in lung cancer mortality. Currently, diagnostic imaging of the thorax is used for early detection of lung cancer using radiological methods, liquid biopsy and, more recently, epigenetic markers.

Research on methods of early lung cancer detection by computed tomography (CT) has provided ambiguous results. In an American study on nearly 55,000 people at high risk of lung cancer it was shown that CT screening resulted in a reduction in mortality by 20% through the use of low-dose CT screening compared with standard chest radiography [2]. In contrast, the results of studies on Europeans with fewer numbers of people at risk of lung cancer gave inconsistent results [3, 4]. Diagnostic imaging can often lead to the misreading of results and result in false positive of lung nodules for which malignancy could not be defined prior to surgery and pathology [4].

The liquid biopsy is another method for early detection of lung cancer. Usually a total blood sample is taken for diagnosis, prognosis, and prediction of therapeutic response [5]. Genetic markers such as circulating cell-free tumor DNA (cfDNA), e.g., *TP53* mutations in the plasma cfDNA from SCLC (squamous cell lung cancer) cases; telomerase reverse transcription (*TERT*); exosomes; tumor-educated platelets (TEP); cell-free tumor RNA (cfRNA); plasma microRNAs (e.g. miR-1254 and miR-574-5p, miR-21, miRNA-126, miR-210, and miR-486-5p) in early-stage NSCLC (non-squamous cell lung cancer); circulating tumor cells (CTCs) [5, 6] and tumor-related antigens-p53, GBU4-5, NY-ESO-1, CAGE, Annexin 1, and SOX2 [7]. These molecular markers are all potentially valuable for prediction but lack rigorous large-scale assessment and as such are not ready for widespread use.

One of the most important examples of early detection is methylation of the *p16INK4a* promoter in DNA derived from bronchoalveolar lavage of resectable NSCLC [8]. Other examples include identification of smokers with the highest risk of lung cancer up to 3 years prior to clinical diagnosis due to the promoter hypermethylation of a panel of seven genes: *CDKN2A*, *PAX5β*, *MGMT*, *DAPK*, *GATA5*, *GATA4*, and *RASSF1A* in sputum [9] or higher methylation frequency in NSCLC compared with normal tissues for 9 genes (*APC*, *CDH13*, *KLK10*, *DLEC1*, *RASSF1A*, *EFEMP1*, *SFRP1*, *RAR-β*, and *P16INK4A*) determined by methylation profiles by MSP (methylation-specific PCR) in tissue and plasma samples [10].

The disadvantage of these methods is the high cost and the fact that they often detect lung pathologies that are not cancer. Therefore, non-invasive and inexpensive screening methods are being sought that could detect early disease in people with the highest probability of developing lung cancer.

Two important factors that influence lung risk include some heavy metals and naturally occurring genetic differences occurring in encoded proteins involved in the removal or amelioration of the effects of the heavy metal [11–13]. Carcinogenic compounds associated with lung cancer include arsenic, asbestos, beryllium, cadmium, chromium, diesel fumes (benzopyrenes), nickel and silica [14].

Exposure to Cr occurs primarily by inhalation in occupational settings. The International Agency for Research on Cancer (IARC) classified chromium(VI) as a carcinogen with sufficient evidence in humans for lung cancer. Except for a few reports from China, little is known about the health risks of environmental exposure to chromium [15]. Chromium is one of the elements widespread in the earth’s crust. This element in small amounts is physiologically necessary by taking part in the metabolism of glucose, certain proteins and fats [16–18].

Mechanism of Cr carcinogenicity is unclear. There are some potential molecular mechanisms for Cr(VI)-associated carcinogenicity including Cr(VI)-induced oxidative stress, intracellular Cr(VI) metabolism, Cr(VI)-induced DNA damage and mutagenesis, and Cr(VI)-induced inflammation-all associated with tumor development [19]. Cr(VI) has been found to be highly mutagenic and penetrates into the cells and is reduced by ascorbate, NADPH, GSH [20, 21], etc., which results in the formation of reactive intermediate forms Cr(IV) and Cr(V) as well as free radicals [22–24].

As a result of Cr(VI) reduction, reactive species appear, such as free radicals, superoxide anions and hydroxyl radicals, probably via a Fenton-like reaction of Cr(V) and Cr(IV) with hydrogen peroxide [25]. A study performed in human epithelial-like L-41 cells and fetal human lung fibroblasts revealed that a toxic Cr(VI) concentration (20 μM) lead to an increase in ROS, and a significant reduction in catalase, glutathione, and cytosolic superoxide dismutase activity [26]. DNA damage as a result of chromium exposure is thought to be the primary mechanism of genotoxicity and mutagenicity. Among the structural genetic changes produced by Cr(VI) are: DNA adducts, DNA strand breaks, DNA-protein crosslinks, oxidized bases, abasic sites, and DNA inter- and intrastrand crosslinks [27]. Evidence suggests that chromium-DNA adducts lead to DNA double strand breaks and inhibition of their repair to cause chromosome instability [28–31]. Genomic instability caused by dysregulated DNA repair mechanism via chromosome instability (CIN), microsatellite instability (MIN) and abnormal cell cycle checkpoints play an important role in Cr(VI) carcinogenesis [32].

The potential role of chromate in carcinogenesis-induced epigenetic alteration is also postulated. For example, in lung cancer samples that had been exposed to chromate, 62.5% had *MLH1* methylation that was correlated with MLH1 repression [33]. In addition, methylation and, consequently, reduced protein expression of the *CDKN2A* has been demonstrated in lung cancer [34]. Some studies hypothesize that Cr(VI) may affect global and promoter-specific histone methylation, leading to gene silencing events [35].

It has been suggested that the development of lung cancer is associated with a proinflammatory state, most often chronic pneumonia especially in non-smokers [36]. A wide range of immune responses are caused by exposure to chromium —essentially inhalation of chromium particles causes damage to lung tissue and an inflammatory response in the lungs [37].

Not all Cr(VI) compounds are carcinogenic, some are cytotoxic and others genotoxic [38]. Relatively insoluble, disintegrated Cr(VI) compounds (such as zinc, lead, strontium, and sintered calcium chromate) show the greatest toxicity leading to transformation in mouse cells and the development of cancer in animals [38, 39]. *In vivo* and cell culture studies have shown an increased incidence of tumor transformation and tumor formation which correlates with workers exposed to certain forms of Cr(VI) who have a significant increase in the risk of lung cancer [40, 41]. Low levels of *p53* mutation, abnormal *p16INK4A* methylation, loss of *MLH1* expression and, consequently, an increase in microsatellite instability can be observed in human lung cancer cells associated with Cr(VI) exposure [33, 34, 42].

It is well established that increased risks of cancer are associated with polymorphisms of different genes. For the studies herein, we selected polymorphisms in genes reported to be directly involved in malignant transformation and/or xenobiotic metabolism and/or oxidative stress (*ERCC2*, *XRCC1*, *MT1B*, *GSTP1*, *ABCB1*, *NQ01*, *CRTC3*, *GPX1*, *SOD2*, and *CAT*). The selected polymorphisms characterized by relatively high frequency critical for association studies were interrogated to determine if they influenced lung cancer risk in association with Cr(VI) exposure.

Direct malignant transformation has been reported as a consequence of changes in *ERCC2* and *XRCC1*. The single nucleotide polymorphism (SNP) rs13181 in *ERCC2* involved in nucleotide excision repair (NER) and carcinogen metabolism, has been associated with lung cancer risk [43]. The results of a meta-analysis strongly implicate *XRCC1* (X-Ray Repair Cross Complementing 1) in cancer development, especially rs1799782 and its association with thyroid cancer [44].

Among the genes encoding proteins involved in xenobiotic metabolism, five are of particular interest: *MT1B*, *GSTP1*, *ABCB1*, *NQ01* and *CRTC3*.

The protein encoded by *MT1B* gene plays a role in metal metabolism and protects cells against the toxic effects of radiation. It is also involved in the regulation of zinc and copper homeostasis, and the polymorphism rs7191779 correlates with the risk for oral squamous cell carcinoma [45]. It was assumed that polymorphisms that change the activity of *GSTP1* would be risk modifiers and markers in the development of lung cancer [46]. The rs1695 SNP in *GSTP1*, implicated in phase II metabolism of many substrates, including xenobiotics, is recognized as a risk factor for lung cancer [47].

*ABCB1* belongs to a superfamily of ATP binding cassette (ABC) transporters, and is also known as *MDR1*. The *ABCB1* polymorphism rs2032582 is associated with differential function of the protein where its potential role in toxic metal secretion or toxicity remain unexplored [48]. *NQO1*-NAD(P)H: quinone oxidoreductase is a flavoenzyme associated with carcinogen metabolism [49]. According to a meta-analysis the *NQO1* rs12915189 polymorphism is associated with lung cancer [50]. *CRTC3* belongs to the family of CREB transcription coactivator genes (a protein binding the cAMP response element) [51]. The SNP rs12915189 in *CRTC3* has been associated with chromium level in humans [52, 53].

Among the genes associated with oxidative stress, we selected polymorphisms within *GPX1*, *SOD2*, and *CAT* genes. *GPX1* is a selenium-dependent enzyme that participates in the detoxification of hydrogen peroxide and a wide range of organic peroxides. It is reported that the *GPX1* polymorphism (Pro198Leu, rs1050450) may contribute significantly to lung cancer risk [54, 55]. Superoxide dismutase 2 (*SOD2*) belongs to the superoxide dismutase family, which can transform toxic superoxide into hydrogen peroxide and diatomic oxygen. The results of a meta-analysis strongly suggest that the rs4880 polymorphism in *SOD2* is significantly associated with the occurrence of lung cancer [56]. *CAT* encodes an enzyme common to all living organisms and is responsible for catalyzing the decomposition of hydrogen peroxide into water and oxygen. It was observed that reduced *CAT* activity caused by inflammation in the lungs can lead to an intracellular increase in hydrogen peroxide and the formation of an intracellular environment suitable for DNA damage and cancer promotion [57]. A meta-analysis on the relationship between the *CAT* polymorphism rs1001179 and cancer risk showed a significant association with the risk of prostate cancer [58].

Our aim was to assess whether serum chromium levels, as well as DNA variants in selected genes involved in carcinogenesis, xenobiotic-metabolism, and oxidative stress could be helpful in the detection of lung cancer.

## Materials and Methods

### Study Group

Two hundred eighteen patients with lung cancer participated in the study and gave informed consent. They were randomly included in this research at the Department of Thoracic Surgery in Szczecin-Zdunowo Hospital between 2012 and 2017. In all patients, lung cancer was confirmed by histopathological examination. The study was conducted in accordance with the Helsinki Declaration and with the consent of the Ethics Committee of Pomeranian Medical University in Szczecin under the number KB-0012/73/10.

Blood samples were taken from patients at the time of diagnosis but before treatment. They were then stored at − 80 °C. For each lung cancer patient one unaffected individual registered at the International Hereditary Cancer Center, Pomeranian Medical University of Szczecin, was matched as a healthy control subject. Control subjects were part of a population-based study of the 1.3 million inhabitants of Poland designed to identify familial aggregations of cancer conducted by our center. All control subjects were enrolled in the study after providing written informed consent. Participants were matched for year of birth (± 3 years), sex, smoking status (pack-years ± 20%) and the total number of lung and other malignancies among first degree relatives. All patients were fasting at least six hours before blood sample collection. The characteristics of the individuals included in the study are shown in Table 1.

**Table 1:** Characteristic of individuals for lung cancer study

Characteristics	Case (*n* = 218)	Control (*n* = 218)
Birth year range	1926–1966	1926–1968
Age at sample, mean (range, year)	63.93 (47–87)	63.59 (48–86)
Sex		
Male	163	163
Female	55	55
Pack-years, mean (range)	34.52 (3–135)	30.43 (2–100)
Smoking status		
Yes	106	106
No	112	112
Stage		
I	71	–
IA	36	–
IA1	4	–
IA2	17	–
IA3	15	–
IB	35	–
II	40	–
IIA	19	–
IIB	21	–
III	85	–
IIIA	45	–
IIIB	35	–
IIIC	5	–
IV	15	–
IVA	11	–
IVB	4	–
Missing	7	–

### Measurement of Cr Level

Total serum chromium levels were measured in the Metals Analysis Laboratory, Nofer Institute of Occupational Medicine. The inductively coupled plasma mass spectroscopy (ICP-MS) technique using NexION 350D (PerkinElmer, USA) was used for sample analysis. Chromium was measured in DRC mode with ammonia (NH3, purity > 0.9999) as a reaction gas for removing spectral interference.

Calibration curve standards (0.1–10.0 μg/L) were prepared and an external calibration method was used. The correlation coefficient of the Cr calibration curve was always greater than 0.999.

The Laboratory participated in an external quality control (G-EQUAS) program to ensure accuracy, using certified/reference standards (ClinCheck® Plasma Control, Recipe, Germany).

### Molecular Analyses

Ten selected variants in ten genes were genotyped: rs13181 in *ERCC2*, rs1799782 in *XRCC1*, rs7191779 in *MT1B*, rs1695 in *GSTP1*, rs2032582 in *ABCB1*, rs1800566 in *NQO1*, rs12915189 in *CRTC3*, rs1050450 in *GPX1*, rs4880 in *SOD2*, and rs1001179 in *CAT*. From each individual included in the study, a 10 mL peripheral blood sample was collected in a vacutainer tube containing 1 mL of 10% sodium EDTA (EthyleneDiamineTetraacetic Acid). The genomic DNA was isolated using the detergent method [59]. SNP analyses were performed using a pre-designed Genotyping Assay × 40 (Applied Biosystems). Each reaction mixture consisted of 2.5-μL LightCycler 480 Probe Master Mix (Roche Diagnostics), the assay 0.125 μL (Genotyping Assay × 40 TaqMan, Applied Biosystems), and 1.375-μL deionized water (Roche Diagnostics). Samples were analyzed on 384-well plates. Each plate was included positive, negative and water-blind control. The genotyping data were collected and analyzed using the LightCycler 480 Instrument and the program of the LightCycler 480 Basic Software Version 1.5 (Roche Diagnostics).

### Statistical Analysis

For the estimation of association of chromium levels or chromium level and genotype with lung cancer occurrence, study participants were assigned to one of four categories (quartiles) based on the chromium distribution in the entire group. The association of chromium levels with lung cancer occurrence was estimated by odds ratio (OR) analysis with 95% confidence intervals using univariable conditional logistic regression. The quartile with the highest amount/ratio of healthy subjects was considered the reference category for the odds ratio calculation.

All calculations were performed in the R statistical environment (R Version 3.6.1 2019-07-05).

## Results

The analysis of lung cancer occurrence based on serum chromium levels of the entire group revealed the highest frequency of cancer in the quartile with the highest chromium levels. The OR difference between the quartile with the highest and the lowest chromium levels (quartile IV vs. quartile II) was 4.52 (Table 2). Differences between sub-groups of smokers and non-smokers were small—OR for the entire group did not differ more than 20% from sub-groups of smokers and non-smokers (Supplementary Material Tables 1 and 2).

**Table 2:** Serum chromium levels and the occurrence of lung cancer

Quartile	Cr level (μg/L)	Cases	Controls	OR (95% CI)	*p-* value
I	0.03–0.07	38	71	1.23 (0.60–2.52)	0.58
II	0.08–0.09	24	50	1.00 (−)	–
III	0.10–0.14	86	55	*3.97 (1.98–7.94)*	< 0.01
IV	0.15–1.63	70	42	*4.52* (*2.17–9.42*)	< 0.01

Italics - Results with statistical significance

The above correlation was not dependent on clinical stage - there were no differences between subgroups of combined stages I and II and stages III with IV (Tables 3 and 4).

**Table 3:** Serum chromium levels and the occurrence of stages I–II of lung cancer

Quartile	Cr level (μg/L)	Cases	Controls	OR (95% CI)	*p-* value
I	0.03–0.07	15	38	1.00 (−)	–
II	0.08–0.10	21	33	1.91 (0.70–5.17)	0.21
III	0.11–0.14	37	16	*6.01* (*2.32–15.54*)	< 0.01
IV	0.15–1.36	38	24	*5.07* (*1.95–13.19*)	< 0.01

Italics - Results with statistical significance

**Table 4:** Serum chromium levels and the occurrence of stages III–IV of lung cancer

Quartile	Cr level (μg/L)	Cases	Controls	OR (95% CI)	*p-* value
I	0.03–0.06	11	24	1.00 (−)	–
II	0.07–0.09	24	32	1.61 (0.67–3.86)	0.29
III	0.10–0.13	31	25	*3.05* (*1.22–7.63*)	0.02
IV	0.14–1.63	34	19	*4.68* (*1.73–12.68*)	< 0.01

Italics - Results with statistical significance

When only stage I disease was compared against Cr levels, the OR of the lowest quartile (quartile 1) against quartile IV was 5.15 with *p* = 0.01, CI 1.5–17.8 and for quartile III OR was 8.72, *p* < 0.01, CI 2.6–29.8 (Table 5).

**Table 5:** Serum chromium levels and the occurrence of stage I of lung cancer

Quartile	Cr level (μg/L)	Cases	Controls	OR (95%CI)	*p-* value
I	0.03–0.07	9	27	1.00 (−)	–
II	0.08–0.10	10	17	2.34 (0.62–8.76)	0.21
III	0.11–0.15	30	11	*8.72* (*2.56–29.76*)	< 0.01
IV	0.16–1.36	22	16	*5.15* (*1.49–17.78*)	0.01

Italics - Results with statistical significance

The results for all stages are available in Supplementary Material Table 3.

Inclusion of genotype data from the ten selected polymorphisms revealed an even greater correlation with disease. Several of the genotypes appeared to be associated with a significantly increased correlation with disease (> 2 times) between serum chromium levels and the probability of lung cancer (Table 6).

**Table 6:** Genotypes and quartiles of Cr level with the highest/lowest frequency of lung cancer *

DNA variant	Cr level (μg/L)–quartile IV	Quartile IV-cases/controls vs. reference quartile-cases/controls		OR (95% CI)	*p* value
*ERCC2*-rs13181 TT	> 0.12	11/4	5/8	*12.34* (*1.17–130.01*)	0.04
*CRTC3-*rs12915189 nonGG	> 0.14	18/9	6/13	*9.73* (*1.58–60.10*)	0.01
*GSTP1*-rs1695 nonAA	> 0.15	21/14	9/22	*9.47* (*2.06–43.49*)	< 0.01
*CAT*-rs1001179 nonCC	> 0.15	16/8	5/16	*9.18* (*1.64–51.24*)	0.01

* Statistical analyses on sub-groups of pairs matched also for genotypes

Italics - Results with statistical significance

Genotypes with the strongest effect included: *ERCC2* rs13181TT with OR 12.34; *CRTC3* rs12915189 nonGG with OR 9.73; and *GSTP1* rs1695 nonAA with OR 9.474; *CAT* rs1001179 nonCC with OR 9.18.

The results for other genotypes are available in Supplementary Material Table 4.

## Discussion

Lung cancer is the leading tumor for mortality worldwide [1]. The occurrence of lung cancer is affected by environmental exposure and genetic or epigenetic susceptibility to disease development and progression [60]. Important factors associated with lung cancer development are occupational exposure to carcinogens (arsenic, asbestos, beryllium, cadmium, chromium, diesel fumes, nickel, and silica) [14]. According to the European Commission, based on socioeconomic, health, and environmental impact assessment, the strongest factors related to attributable cancer deaths include Cr(VI) [61]. However, this is only an estimate on the impact of occupational exposure and not based on general population data.

The toxicity of chromium is highly dependent on its chemical form. Very high levels of serum ascorbate lead to the rapid reduction of Cr(VI) to Cr(III) thus, Cr(VI) levels in humans that have high levels of ascorbate would be expected to be low. Furthermore, Cr(VI) reduction would be expected to occur at or near the site of exposure (lung or gastrointestinal tract), which results in low circulating blood levels of Cr(VI). Taking this into account, total Cr levels are the appropriate measurement for this study and it is justified not to assay different Cr species.

In the general population, the mean levels of Cr in serum and urine are 0.10–0.16 and 0.22 μg/L, respectively [62]. The mean total chromium levels in both groups of people diagnosed with lung cancer and controls were within the limits proposed by ATSDR (Agency for Toxic Substances and Disease Registry), i.e. they do not deviate from the values considered normal for the general population. Although the chromium levels in the group of patients with cancer are statistically significantly higher compared with those in the control group (*p* < 0.05), they were still within the normal range [62].

The data herein suggests that it may be attractive for practical purposes. We plan to measure serum chromium levels in patients from our cancer genetic outpatient clinics especially for subgroups of individuals with familial lung and/or other cancer aggregations. Patients from such families having high serum chromium levels will be provided with the option of surveillance CT scans of the lung for early disease detection. Analyses of chromium levels may be an attractive option to identify patients with early stage disease especially because the frequency of lung cancer is significantly increased (OR > 5) if the serum chromium level is above 0.1 μg/L. Additionally, it is very interesting that total serum chromium levels > 0.1 μg/L appear to be associated with 73% (52/71) of lung cancer patients with stage I disease. It is well recognized that lung cancer treatment success is correlated with the clinical stage at diagnosis. De Matteis et al. studied the effect of carcinogens on the risk of lung cancer in the general population. They showed that patients appear to have an increased risk of lung cancer due to exposure to nickel-chromium (OR 1.18; 95% CI 0.90–1.53), which is consistent with our study results [14]. Since smoking is one of the most important risk factors for lung cancer, we included this factor in our regression model. Differences between smoking and non-smoking subgroups were small—with the OR for the whole group not differing by more than 20% between smoking and non-smoking groups (Supplementary Materials, Tables 1 and 2). This is in contradiction with the work of others, since it has been reported that chromium levels are much higher in smokers’ lung tissues than in those of non-smokers [63].

Moreover, in our studies, we have been able to show that functional DNA variants in some genes involved in carcinogenesis, oxidative stress and xenobiotics clearance probably enhance the effects of chromium. Variants associated with an increased risk of lung cancer included *ERCC2*, *CRTC3*, *GSTP1*, and *CAT.* Generally, except for CRTC3 rs12915189, SNPs selected for this study were not recognized as affecting Cr levels in serum. The most likely explanation concerning these polymorphisms is that they modify the physiological response occasioned by the presence of Cr(VI). Further investigation is needed to explain in more detail the mechanisms of action between the effects of Cr and the functions of the respective genes. In our series, no single polymorphism was by itself altering the probability of lung cancer occurrence (Supplementary Material Table 5).

The data concerning sub-groups of particular genotypes were achieved on smaller number of patients and should be taken with special caution. Further investigations validating our results are required on larger groups of patients from different geographic regions and ethnic groups.

Another limitation of our study is that, the high risk of lung cancer is generally recognized as being associated with occupational exposure to Cr(VI), but we did not have patient work histories available to include in this study. Nevertheless, our study may provide an avenue to begin to screen for lung cancer occurrence, which is based on the large sample size and analyses focused on the general population.

## Conclusion

In summary, our research provides evidence to connect Cr exposure to an increased incidence of lung cancers. However, these findings require the support by future studies that are capable of addressing the problem of other potential confounders in the association between exposure to Cr(VI) and lung cancer in the general population. At this time, we suggest that analysis of serum total chromium after further investigations on the medical and cost-effectiveness of this approach, might be useful in the effective detection of early lung cancers, especially in individuals with special genotypes.

## Electronic Supplementary Material

ESM 1 (PDF 43.7 kb)
