# Supplementary material for: Lung Cancer Occurrence—Correlation with Serum Chromium Levels and Genotypes
Source: Biol Trace Elem Res. 2020 Jul 9;199(4):1228–36. doi: 10.1007/s12011-020-02240-6 (PMC7886837; doi:10.1007/s12011-020-02240-6)
Supplement: Supplementary file 1 — (PDF 43.7 kb) [file 12011_2020_2240_MOESM1_ESM.pdf]

**Supplementary Material Table1. Serum chromium levels and the occurrence of lung cancer among smokers**

| Quartile | Cr level [µg/L] | Cases | Controls | OR | CI | p-value |
|---|---|---|---|---|---|---|
| I | 0.04-0.07 | 18 | 34 | 1.00 | - | - |
| II | 0.08-0.10 | 19 | 33 | 1.17 | 0.47-2.91 | 0.74 |
| III | 0.11-0.14 | 33 | 18 | **3.23** | 1.36-7.65 | <0.01 |
| IV | 0.15-0.95 | 36 | 21 | **3.66** | 1.50-8.94 | <0.01 |

**Supplementary Material Table2. Serum chromium levels and the occurrence of lung cancer among non-smokers**

| Quartile | Cr level [µg/L] | Cases | Controls | OR | CI | p-value |
|---|---|---|---|---|---|---|
| I | 0.03-0.06 | 11 | 28 | 1.00 | - | - |
| II | 0.07-0.09 | 20 | 37 | 1.15 | 0.46-2.89 | 0.76 |
| III | 0.10-0.13 | 39 | 22 | **5.06** | 1.96-13.02 | <0.01 |
| IV | 0.14-1.63 | 42 | 25 | **5.30** | 2.02-13.90 | <0.01 |

**Supplementary Material Table3. Lung cancer occurrence – correlation with serum Cr levels and stages.**

| | Quarter | Cr level (μg/L) | Case | Control | OR | 95%CI | p-value |
|---|---|---|---|---|---|---|---|
| Stage | | | | | | | |
| Stage I (n=142) | I | 0.03-0.07 | 9 | 27 | 1.00 | - | - |
| | II | 0.08-0.10 | 10 | 17 | 2.34 | 0.62-8.76 | 0.21 |
| | III | 0.11-0.15 | 30 | 11 | **8.72** | 2.56-29.76 | <0.01 |
| | IV | 0.16-1.36 | 22 | 16 | **5.15** | 1.49-17.78 | 0.01 |
| Stage II (n=80) | I | 0.03-0.07 | 6 | 11 | 1.57 | 0.24-10.35 | 0.64 |
| | II | 0.08-0.09 | 6 | 13 | 1.00 | - | - |
| | III | 0.10-0.13 | 15 | 8 | **7.85** | 1.25-49.20 | 0.03 |
| | IV | 0.14-0.89 | 13 | 8 | **5.49** | 0.98-30.83 | 0.05 |
| Stage III (n=170) | I | 0.03-0.06 | 10 | 21 | 1.00 | - | - |
| | II | 0.07-0.09 | 22 | 27 | 1.71 | 0.69-4.27 | 0.25 |
| | III | 0.10-0.13 | 25 | 21 | **2.75** | 1.03-7.30 | 0.04 |
| | IV | 0.14-0.71 | 28 | 16 | **4.11** | 1.45-11.65 | <0.01 |
| Stage IV (n=30) | I | 0.04-0.07 | 1 | 4 | 1.00 | - | - |
| | II | 0.08-0.10 | 2 | 6 | 1.00 | 0.06-15.99 | 1.00 |
| | III | 0.11-0.13 | 6 | 2 | 1365934879.43 | 0.00-Inf | 1.00 |
| | IV | 0.14-1.63 | 6 | 3 | 2731869758.87 | 0.00-Inf | 1.00 |
| Stage I-II (n=222) | I | 0.03-0.07 | 15 | 38 | 1.00 | - | - |
| | II | 0.08-0.10 | 21 | 33 | 1.91 | 0.70-5.17 | 0.21 |
| | III | 0.11-0.14 | 37 | 16 | **6.01** | 2.32-15.54 | <0.01 |
| | IV | 0.15-1.36 | 38 | 24 | **5.07** | 1.95-13.19 | <0.01 |
| Stage III-IV (n=200) | I | 0.03-0.06 | 11 | 24 | 1.00 | - | - |
| | II | 0.07-0.09 | 24 | 32 | 1.61 | 0.67-3.86 | 0.29 |
| | III | 0.10-0.13 | 31 | 25 | **3.05** | 1.22-7.63 | 0.02 |
| | IV | 0.14-1.63 | 34 | 19 | **4.68** | 1.73-12.68 | <0.01 |

**Supplementary Material Table4. Lung cancer occurrence – correlation with serum Cr levels and genotypes.**

| | Quarter | Cr level (μg/L) | Case | Control | OR | 95%CI | p-value |
|---|---|---|---|---|---|---|---|
| Genotype | | | | | | | |
| *CAT* CC (n=164) | I | 0.04-0.07 | 13 | 23 | 1.00 | - | - |
| | II | 0.08-0.09 | 12 | 21 | 0.97 | 0.34-2.72 | 0.95 |
| | III | 0.10-0.13 | 28 | 19 | **2.89** | 1.10-7.60 | 0.03 |
| | IV | 0.14-1.36 | 29 | 19 | **2.83** | 1.10-7.27 | 0.03 |
| *CAT* nonCC (n=88) | I | 0.04-0.07 | 5 | 16 | 1.00 | - | - |
| | II | 0.08-0.10 | 10 | 12 | 2.93 | 0.48-17.91 | 0.24 |
| | III | 0.11-0.14 | 13 | 8 | **8.11** | 1.36-48.52 | 0.02 |
| | IV | 0.15-0.89 | 16 | 8 | **9.18** | 1.64-51.24 | 0.01 |
| *NQO1* GG (n=228) | I | 0.03-0.07 | 20 | 33 | 1.04 | 0.45-2.38 | 0.94 |
| | II | 0.08-0.10 | 21 | 40 | 1.00 | - | - |
| | III | 0.11-0.14 | 36 | 20 | **2.90** | 1.38-6.08 | <0.01 |
| | IV | 0.15-1.63 | 37 | 21 | **3.68** | 1.60-8.49 | <0.01 |
| *NQO1* nonGG (n=34) | I | 0.04-0.06 | 2 | 4 | 0.75 | 0.05-10.40 | 0.83 |
| | II | 0.07-0.09 | 2 | 6 | 1.00 | - | - |
| | III | 0.11-0.14 | 6 | 4 | 2.54 | 0.32-19.93 | 0.37 |
| | IV | 0.15-0.95 | 7 | 3 | 5.86 | 0.59-58.40 | 0.13 |
| *MT1B* GG (n=50) | I | 0.06-0.08 | 6 | 7 | 1.55 | 0.34-7.07 | 0.57 |
| | II | 0.09-0.11 | 3 | 6 | 1.00 | - | - |
| | III | 0.12-0.16 | 7 | 6 | 2.58 | 0.46-14.63 | 0.28 |
| | IV | 0.17-0.75 | 9 | 6 | 4.00 | 0.57-28.26 | 0.16 |
| *MT1B* nonGG (n=176) | I | 0.03-0.06 | 8 | 24 | 1.00 | - | - |
| | II | 0.07-0.09 | 20 | 30 | 2.31 | 0.77-6.93 | 0.14 |
| | III | 0.10-0.14 | 29 | 20 | **4.76** | 1.60-14.12 | <0.01 |
| | IV | 0.15-1.36 | 31 | 14 | **7.23** | 2.28-22.93 | <0.01 |
| *CRTC3* GG (n=128) | I | 0.03-0.07 | 13 | 16 | 2.43 | 0.73-8.12 | 0.15 |
| | II | 0.08-0.10 | 10 | 25 | 1.00 | - | - |
| | III | 0.11-0.14 | 21 | 9 | **5.44** | 1.71-17.31 | <0.01 |
| | IV | 0.15-0.68 | 20 | 14 | **3.91** | 1.22-12.54 | 0.02 |
| *CRTC3* nonGG (n=106) | I | 0.04-0.07 | 12 | 15 | 2.23 | 0.39-12.70 | 0.37 |
| | II | 0.08-0.09 | 6 | 13 | 1.00 | - | - |
| | III | 0.10-0.13 | 17 | 16 | 4.74 | 0.88-25.53 | 0.07 |
| | IV | 0.14-0.89 | 18 | 9 | **9.73** | 1.58-60.10 | 0.01 |
| *GPX1* CC (n=110) | I | 0.03-0.07 | 9 | 19 | 1.00 | - | - |
| | II | 0.08-0.10 | 8 | 11 | 1.32 | 0.35-5.02 | 0.68 |
| | III | 0.11-0.14 | 17 | 14 | 2.64 | 0.92-7.53 | 0.07 |
| | IV | 0.15-0.75 | 21 | 11 | **4.23** | 1.34-13.34 | 0.01 |
| *GPX1* nonCC (n=122) | I | 0.04-0.06 | 6 | 15 | 1.00 | - | - |
| | II | 0.07-0.09 | 11 | 23 | 1.30 | 0.41-4.13 | 0.66 |
| | III | 0.10-0.14 | 22 | 12 | **5.75** | 1.45-22.76 | 0.01 |
| | IV | 0.15-1.63 | 22 | 11 | **6.72** | 1.65-27.38 | <0.01 |
| *SOD2* GG | I | 0.04-0.08 | 4 | 4 | 1.85 | 0.16-21.67 | 0.62 |

| | | | | | | | |
|---|---|---|---|---|---|---|---|
| (n=30) | II | 0.09-0.10 | 1 | 2 | 1.00 | - | - |
| | III | 0.11-0.17 | 6 | 5 | 2.35 | 0.16-33.69 | 0.53 |
| | IV | 0.18-1.63 | 4 | 4 | 2.09 | 0.09-47.20 | 0.64 |
| *SOD2* nonGG (n=220) | I | 0.03-0.07 | 15 | 36 | 1.00 | - | - |
| | II | 0.08-0.10 | 20 | 37 | 1.45 | 0.57-3.69 | 0.44 |
| | III | 0.11-0.14 | 37 | 17 | **5.79** | 2.23-15.05 | <0.01 |
| | IV | 0.15-1.36 | 38 | 20 | **6.06** | 2.22-16.51 | <0.01 |
| *XRCC1* CC (n=336) | I | 0.04-0.07 | 28 | 53 | 1.35 | 0.58-3.15 | 0.49 |
| | II | 0.08-0.09 | 20 | 44 | 1.00 | - | - |
| | III | 0.10-0.13 | 58 | 35 | **4.49** | 2.00-10.12 | <0.01 |
| | IV | 0.14-0.95 | 62 | 36 | **4.82** | 2.16-10.72 | <0.01 |
| *ERCC2* TT (n=52) | I | 0.03-0.06 | 5 | 8 | 1.28 | 0.22-7.29 | 0.78 |
| | II | 0.07-0.09 | 4 | 9 | 1.00 | - | - |
| | III | 0.10-011 | 6 | 5 | 2.43 | 0.50-11.84 | 0.27 |
| | IV | 0.12-0.75 | 11 | 4 | **12.34** | 1.17-130.01 | 0.04 |
| *ERCC2* nonTT (n=194) | I | 0.03-0.07 | 12 | 23 | 1.00 | - | - |
| | II | 0.08-0.10 | 21 | 30 | 1.42 | 0.54-3.74 | 0.48 |
| | III | 0.11-0.14 | 34 | 21 | **3.01** | 1.21-7.51 | 0.02 |
| | IV | 0.15-1.63 | 30 | 23 | **2.75** | 1.03-7.34 | 0.04 |
| *ABCB1* CC (n=48) | I | 0.03-0.06 | 3 | 6 | 1.00 | - | - |
| | II | 0.07-0.09 | 6 | 8 | 1.73 | 0.28-10.71 | 0.56 |
| | III | 0.10-0.12 | 5 | 6 | 2.54 | 0.36-17.94 | 0.35 |
| | IV | 0.13-0.62 | 10 | 4 | 4.96 | 0.71-34.57 | 0.11 |
| *ABCB1* nonCC (n=202) | I | 0.03-0.06 | 14 | 23 | 1.10 | 0.45-2.69 | 0.84 |
| | II | 0.07-0.09 | 18 | 35 | 1.00 | - | - |
| | III | 0.10-0.14 | 37 | 24 | **4.15** | 1.68-10.25 | <0.01 |
| | IV | 0.15-1.63 | 32 | 19 | **4.81** | 1.83-12.64 | <0.01 |
| *GSTP1* AA (n=90) | I | 0.03-0.07 | 10 | 10 | 3.25 | 0.78-13.57 | 0.11 |
| | II | 0.08-0.09 | 5 | 15 | 1.00 | - | - |
| | III | 0.10-0.13 | 18 | 9 | **8.85** | 1.72-45.57 | <0.01 |
| | IV | 0.14-1.63 | 12 | 11 | 3.12 | 0.73-13.43 | 0.13 |
| *GSTP1* nonAA (n=132) | I | 0.03-0.07 | 9 | 22 | 1.00 | - | - |
| | II | 0.08-0.10 | 16 | 17 | 3.58 | 0.92-13.84 | 0.07 |
| | III | 0.11-0.14 | 20 | 13 | **6.61** | 1.69-25.75 | <0.01 |
| | IV | 0.15-0.75 | 21 | 14 | **9.47** | 2.06-43.49 | <0.01 |

**Supplementary Material Table5. Frequency of genotype prevalence (%) in subjects under observation*.**

| Genotype | Number of cases with genotype/ all cases | Frequency in cases | Number of controls with genotype/ all controls | Frequency in controls | OR | 95%CI | p-value |
|---|---|---|---|---|---|---|---|
| *CAT*-rs1001179 CC | 123/218 | 56.42 | 133/218 | 61.01 | 1.21 | 0.81-1.80 | 0.38 |
| *CAT*-rs1001179 nonCC | 95/218 | 43.58 | 85/218 | 38.99 | | | |
| *NQO1*-rs1800566 GG | 158/218 | 72.48 | 157/218 | 72.02 | 0.98 | 0.63-1.52 | 1.00 |
| *NQO1*-rs1800566 nonGG | 60/218 | 27.52 | 61/218 | 27.98 | | | |
| *MT1B*-rs7191779 GG | 82/218 | 37.61 | 73/218 | 33.49 | 0.84 | 0.55-1.26 | 0.42 |
| *MT1B*-rs7191779 nonGG | 136/218 | 62.39 | 145/218 | 66.51 | | | |
| *CRTC3*-rs12915189 GG | 115/218 | 52.75 | 114/218 | 52.29 | 0.98 | 0.66-1.46 | 1.00 |
| *CRTC3*-rs12915189 nonGG | 103/218 | 47.25 | 104/218 | 47.71 | | | |
| *GPX1*-rs1050450 CC | 105/218 | 48.17 | 107/218 | 49.08 | 1.04 | 0.70-1.54 | 0.92 |
| *GPX1*-rs1050450 nonCC | 113/218 | 51.83 | 111/218 | 50.92 | | | |
| *SOD2*-rs4880 GG | 64/218 | 29.36 | 59/218 | 27.06 | 0.89 | 0.57-1.39 | 0.67 |
| *SOD2*-rs4880 nonGG | 154/218 | 70.64 | 159/218 | 72.94 | | | |
| *XRCC1*-rs17997822 CC | 198/218 | 90.83 | 186/218 | 85.32 | 0.59 | 0.31-1.10 | 0.10 |
| *XRCC1*-rs1799782 nonCC | 20/218 | 9.17 | 32/218 | 14.68 | | | |
| *ERCC2*-rsrs13181 TT | 68/218 | 31.19 | 79/218 | 36.24 | 0.80 | 0.52-1.21 | 0.31 |
| *ERCC2*-rs13181 nonTT | 150/218 | 68.81 | 139/218 | 63.76 | | | |
| *ABCB1*-rs2032582 CC | 73/218 | 33.49 | 68/218 | 31.19 | 0.90 | 0.59-1.37 | 0.68 |
| *ABCB1*-rs2032582 nonCC | 145/218 | 66.51 | 150/218 | 68.81 | | | |
| *GSTP1*-rs1695 AA | 99/218 | 45.41 | 98/218 | 44.95 | 0.98 | 0.66-1.45 | 1.00 |
| *GSTP1*-rs1695 nonAA | 119/218 | 54.59 | 120/218 | 55.05 | | | |

*****No significant differences between cases and controls
